# Arthroscopically Assisted Fixation of Terrible Triad Variant Injuries of the Elbow With Small-Bore Needle Arthroscopy

**DOI:** 10.1016/j.eats.2021.02.011

**Published:** 2021-05-10

**Authors:** Matt Fournier, Evan Corning, Austin Witt, Sarah Lang, Brian B. Gilmer

**Affiliations:** a Tahoe Orthopedic and Sports Medicine, South Lake Tahoe, California, U.S.A.; b Mammoth Orthopedic Institute, Mammoth Hospital, Mammoth Lakes, California, U.S.A.

## Abstract

The range of diagnostic and therapeutic applications of needle arthroscopy (NA) continue to expand due to advances in image quality and resolution. Minimally invasive techniques can be augmented by the smaller camera size and reduced fluid use made possible by NA. Small-bore arthroscopy presents opportunities for use in smaller joints, such as the elbow, where applications of standard arthroscopic equipment may be limited by small anatomic spaces and fluid extravasation. In this Technical Note, we present our technique for NA-assisted treatment of terrible triad injuries, specifically in the setting of an intact radial head. The technique describes a stepwise approach to arthroscopically aided fixation of the coronoid process, followed by open reconstruction of the lateral collateral ligament complex.

Concomitant coronoid, collateral ligament, and radial head injuries in the setting of an elbow instability event traditionally have been considered difficult clinical entities to treat, as evidenced by the name “terrible triad” adopted throughout the literature. Coronoid fractures are especially difficult to address when the radial head component of the injury does not require open treatment, as in nondisplaced fractures or articular injuries involving less and 25% of the articular surface.

Although good outcomes with traditional arthroscopy have been described in the treatment of these injuries, the technique is challenging owing to the proximity of neurovascular structures to the surgical field and the small anatomic spaces at play in the elbow. Despite these challenges, arthroscopic assistance can lead to better visualization of the injuries, reduced surgical morbidity, and lower risk of wound-related complications. Needle arthroscopy (NA) has been described in the diagnostic and therapeutic evaluation of the knee, shoulder, and elbow.^1^, ^2^, ^3^ In this Technical Note, we describe our technique for NA-assisted fixation of a coronoid fracture with an intact radial head and neck. Potential benefits and disadvantages are reviewed.

## Surgical Technique (With Video Illustration)

The surgical technique is demonstrated in Video 1.

### Patient Positioning and Setup

A manual examination with the patient under anesthesia can confirm the typical posterolateral instability pattern by recreating an axial load, valgus, and posterior lateral rotatory force. This is critical for distinguishing an isolated coronoid fracture from a true instability pattern if the preoperative imaging and examination are in question. Furthermore, an intact radial head or neck in the setting of lateral ligament disruption and coronoid fracture should alert the treating surgeon to be aware of osteochondral fracture or loose bodies in the joint, as this can occur as the intact radial head and neck dislocate. If present, these injuries can be treated arthroscopically or through an open lateral approach during ligamentous repair.

General anesthesia is recommended, as regional anesthesia does not allow postoperative assessment of nerve function and patients may not tolerate the positioning while awake. The patient is positioned in the lateral decubitus position on an inflatable bean bag with the arm over a radiolucent arm board. Fluoroscopy can be positioned from the patient’s head with the c-arm and receiver parallel to the side of the bed to allow for ease of intraoperative imaging on the lateral view. A sterile tourniquet is applied after the limb is prepped and draped in the usual fashion and the relevant anatomic landmarks are marked with a sterile marking pen.

### Diagnostic Arthroscopy and Associated Procedures

**Fig 1:**
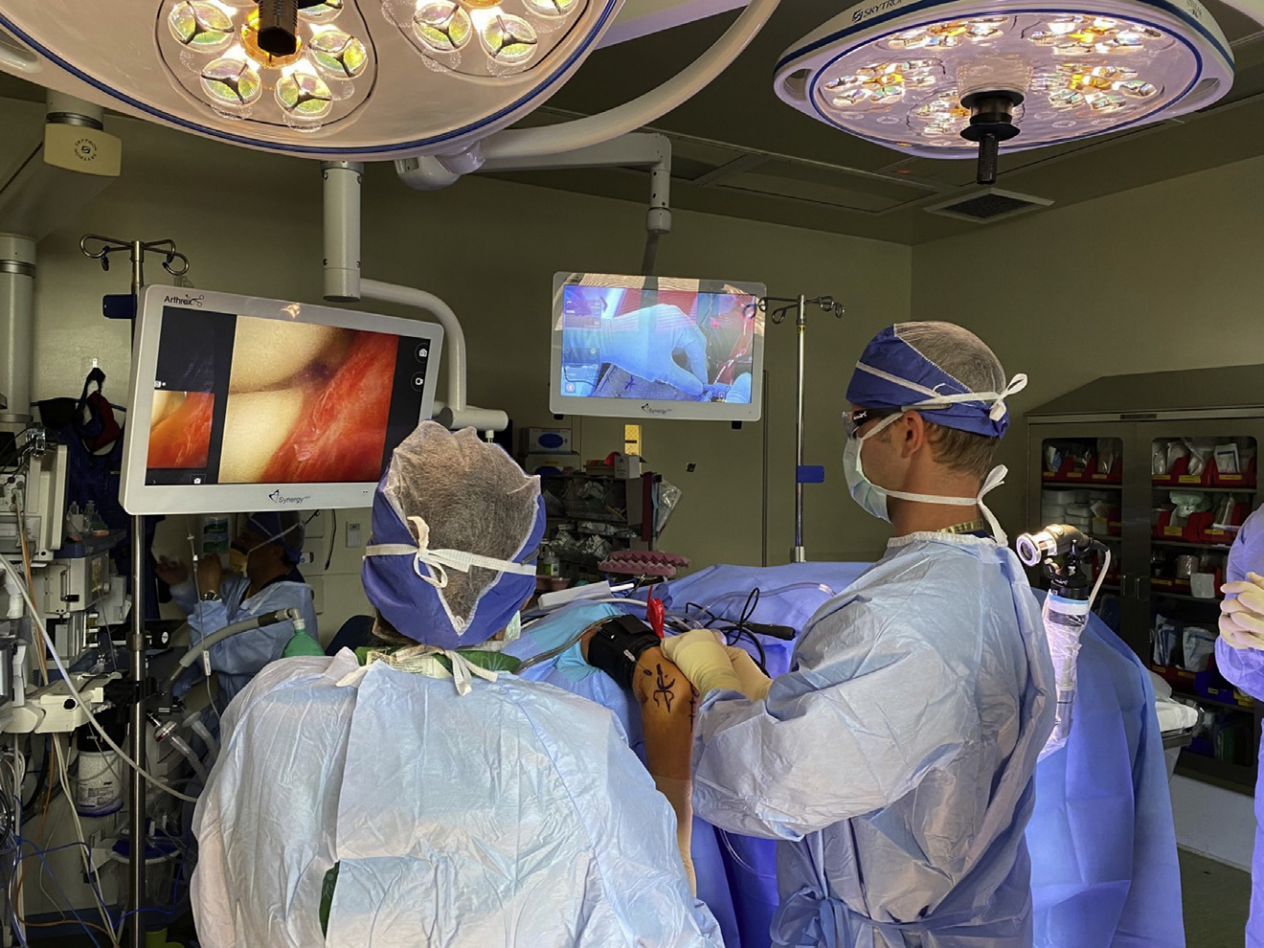
Room setup and patient positioning. The patient is positioned in the right lateral decubitus position on a bean bag, with a radiolucent arm post and a sterile tourniquet applied to the left arm. The arthroscopic monitor is over the patient’s shoulder and the fluoroscopy unit approaches parallel to the bed from the patient’s head.

The NA system (NanoScope; Arthrex, Naples, FL) includes a zero-degree arthroscope with power cord, monitor, and sharp and blunt trocars with corresponding sheaths including inflow portals. Assorted instruments, including a retractable probe and a 2.0-mm shaver, are also available. With the use of a sterile technique, the cords are attached, and the monitor can be relayed to overhead monitors in the operating room via a standard high-definition multimedia interface cable (Fig 1).

**Fig 2:**
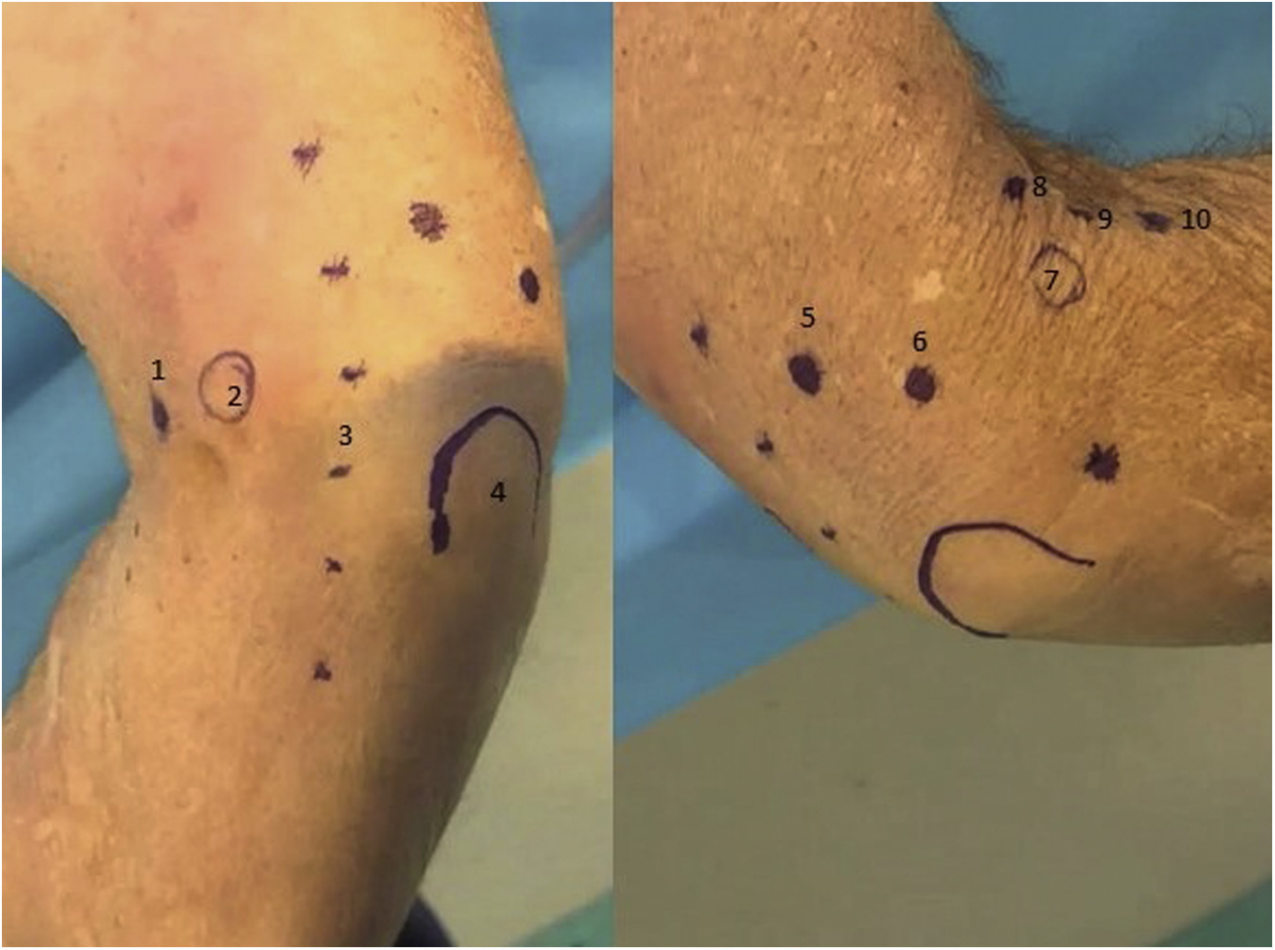
Anatomic landmarks for diagnostic needle arthroscopy of the elbow in a cadaveric specimen are shown. (A) Medial external view of a right elbow 1 – modified proximal anteromedial portal, 2 – medial epicondyle, 3 – ulnar nerve, 4 – olecronon. (B) Lateral external view of a right elbow 5 – transtriceps portal, 6 – posterolateral portal, 7 – lateral epicondyle, 8 – proximal anterolateral portal, 9 – mid-anterolateral portal, and 10 – distal anterolateral portal.

The technique for diagnostic elbow arthroscopy with the NA system has been previously described in detail.^3^ The olecranon, medial epicondyle, lateral epicondyle, and ulnar nerve are identified and marked, as well as the following described portals. For access to the anterior elbow, a modified proximal anteromedial portal (mPAMP), proximal anterolateral portal (PALP), mid-anterolateral portal (MALP), and distal anterolateral portal are used. The mPAMP is used as the primary viewing portal and is located 0.5 to 1 cm proximal to the medial epicondyle and immediately anterior to the intermuscular septum. This is a modification of the classic PAMP position, which is located 2 cm proximal to the medial epicondyle and anterior the intermuscular septum. This modification allows for a better inline view of the entire joint and is necessary due to the zero-degree viewing angle of the NA camera. Despite moving the portal slightly more distal, the decreased size of the NA camera sheath (2 mm vs 4-5 mm) makes injury to the median antebrachial cutaneous nerve unlikely. The PALP functions as the inflow portal and is located 2 cm proximal to the lateral epicondyle and 1 cm anterior to the humerus. The MALP is the primary working portal and provides direct inline access to the joint. It is located 1 cm proximal and 1 cm anterior to the lateral epicondyle. The distal anterolateral portal is used as an accessory working portal and is located 1 to 2 cm distal and 1 cm anterior to the lateral epicondyle, just anterior to the radial head (Fig 2).

### Arthroscopic Fracture Debridement and Reduction

**Fig 3:**
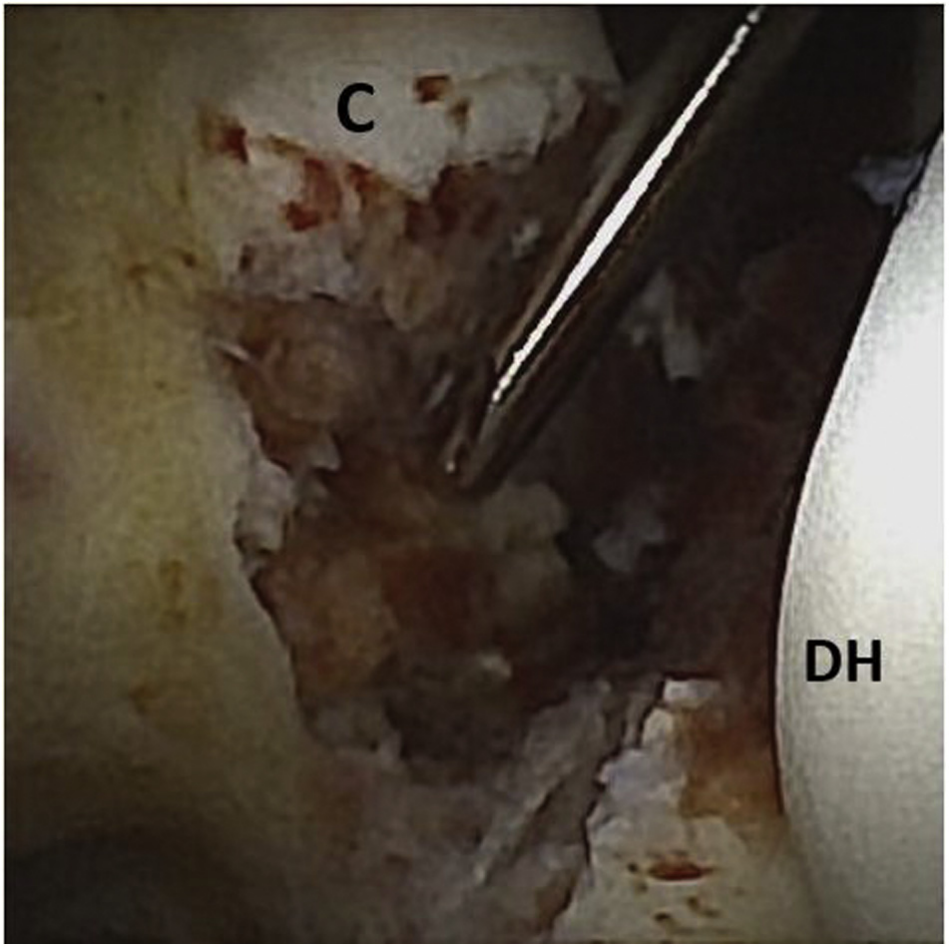
Arthroscopic view, left elbow. View from proximal anterolateral portal. A 2.0 shaver is entering the modified proximal anteromedial portal debriding the fracture bed at the coronoid base. The distal humerus (DH) is to the right, and the coronoid tip (C) is seen superior to the shaver tip.

After completion of a diagnostic arthroscopy, attention is turned to the arthroscopic fracture reduction. The camera with high flow cannula (Arthrex) is placed in the mPAMP and the 2.0 shaver (Saber; Arthrex) is used to debride the fracture bed from the MALP. If the high-flow cannula is not used, inflow from a separate sheath may be provided from the PALP. Use of a small-diameter shaver facilitates fluid management and prevents collapse of the anterior capsule into the field of view by minimizing applied suction (Fig 3). Once adequate debridement is achieved, a provisional fracture reduction can be performed with the assistance of a Kirschner wire (k-wire) or dental pick.

### Open Lateral Dissection

Attention is then turned to the open lateral approach. A standard incision is made from the lateral epicondyle toward the lateral margin of the radial head. Deep dissection typically exploits a traumatic tear in the lateral ligaments; however, if necessary, the Kocher interval between the extensor carpi ulnaris and the anconeus can be used in the rare instance the overlying extensor origin is intact. It is important to remember the proximity of the posterior interosseous nerve and pronate the forearm, which moves the nerve farther from the plane of surgical dissection. In addition, anterior dissection should be performed carefully, as the radial nerve is just anterior to the anterior lateral capsule. Ligamentous tissue most commonly avulses as a sleeve with the common extensor wad. After splitting the interval, the soft tissue is elevated from the lateral epicondyle to provide optimal exposure and a working arthrotomy. A self-retainer is left in place to maintain the open lateral exposure during suture passage and button fixation.

### Passage of Suture Button

**Fig 4:**
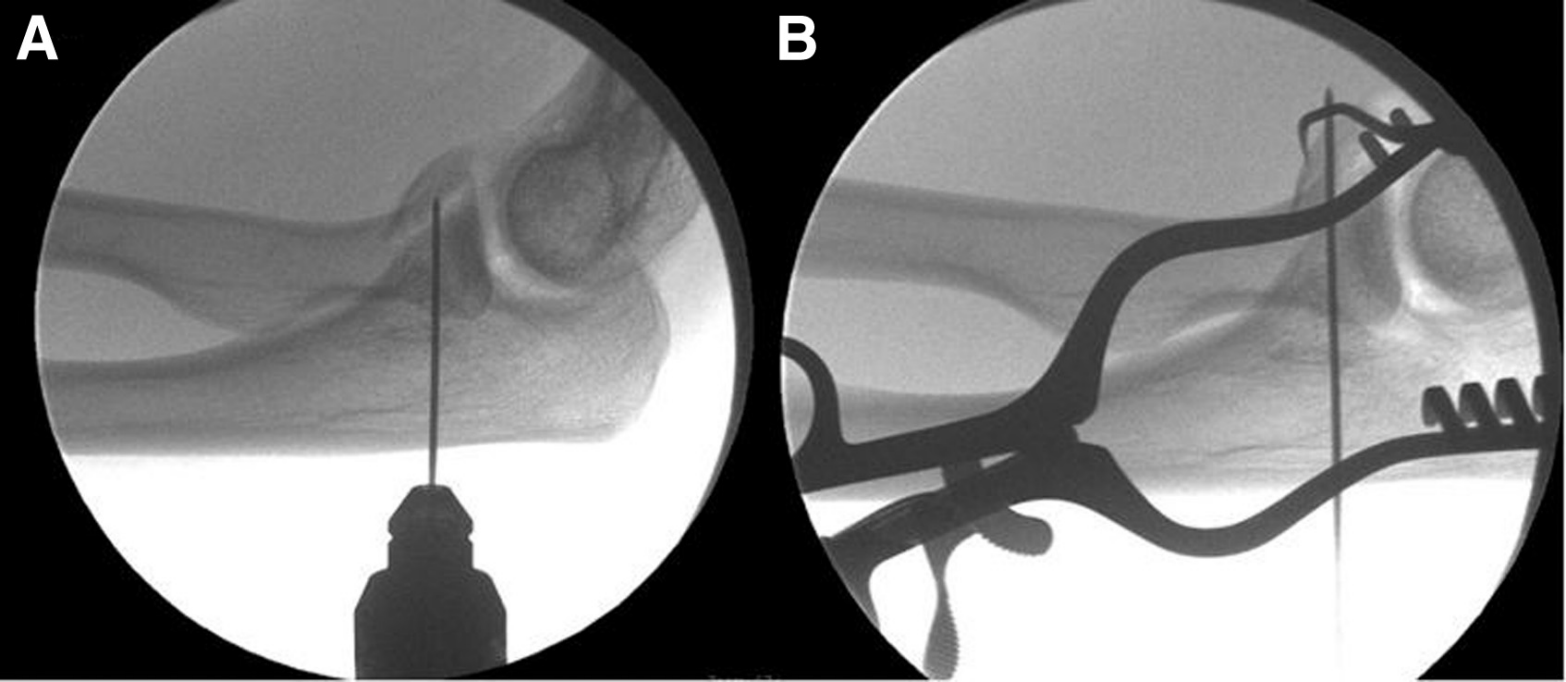
Lateral intraoperative fluoroscopic image of left elbow with (A) guidewire being inserted from the proximal ulna into the fracture bed of the coronoid. (B) Fracture reduction is maintained through with the assistance of a dental pick while the guidewire is passed through the fragment. A self-retaining retractor maintains exposure of the lateral arthrotomy for retrieval of the guidewire.

Under fluoroscopic guidance, a small longitudinal incision is placed on the dorsal border of the proximal ulna in line with the coronoid fracture bed. A guidewire is introduced that contains a wire loop for suture passage. Under direct visualization and while maintaining the reduction with a dental pick or k-wire, the wire is advanced through the coronoid tip fragment. An arthroscopic grasper is then used to retrieve the wire through the open lateral arthrotomy. Position of the wire and fracture reduction are verified fluoroscopically (Fig 4).

A looped end of a passing suture (FiberSnare; Arthrex) is then loaded on the wire loop on the guidewire and retrieved through the lateral portal as the guide wire is removed. This suture is then used to shuttle the 2 tails of a nonabsorbable suture (FiberWire; Arthrex), which are loaded through a 2.6-mm suture button (BicepsButton; Arthrex). As the suture tails are placed in traction through the fracture site and exiting on the dorsal aspect of the proximal ulna, the button is drawn against the coronoid tip effectively reducing the fracture. The reduction is confirmed arthroscopically and fluoroscopically.

One to two centimeters distal to the exiting suture tails a drill guide is placed and a 2.7-mm guide hole is drilled for a 3.5-mm knotless suture anchor (SwiveLock; Arthrex). Suture tails are loaded into the anchor, maximally tensioned, and the anchor is inserted into the bone. Remaining suture tails are cut and discarded. The knotless suture anchor is preferred to a suture button because there is no prominence that may result in irritation on the subcutaneous border of the ulna. The final coronoid reduction is confirmed fluoroscopically.

### Lateral Collateral Ligament Repair

Two suture anchors are then placed on the lateral epicondyle. The first is placed just distal and anterior to the central portion of the anatomic origin of the lateral collateral ligament complex. This is used primarily for fixation of the ligamentous tissue itself. The second is placed more proximally for repair of the common extensor origin that was previously elevated. Repair of the ligaments is performed with horizontal mattress sutures from the anchors which are used to imbricate the anterior flap over the more posterior tissue sleeve. This has the effect of tightening the lateral collateral ligament complex, which often has been attenuated from the initial trauma.

**Fig 5:**
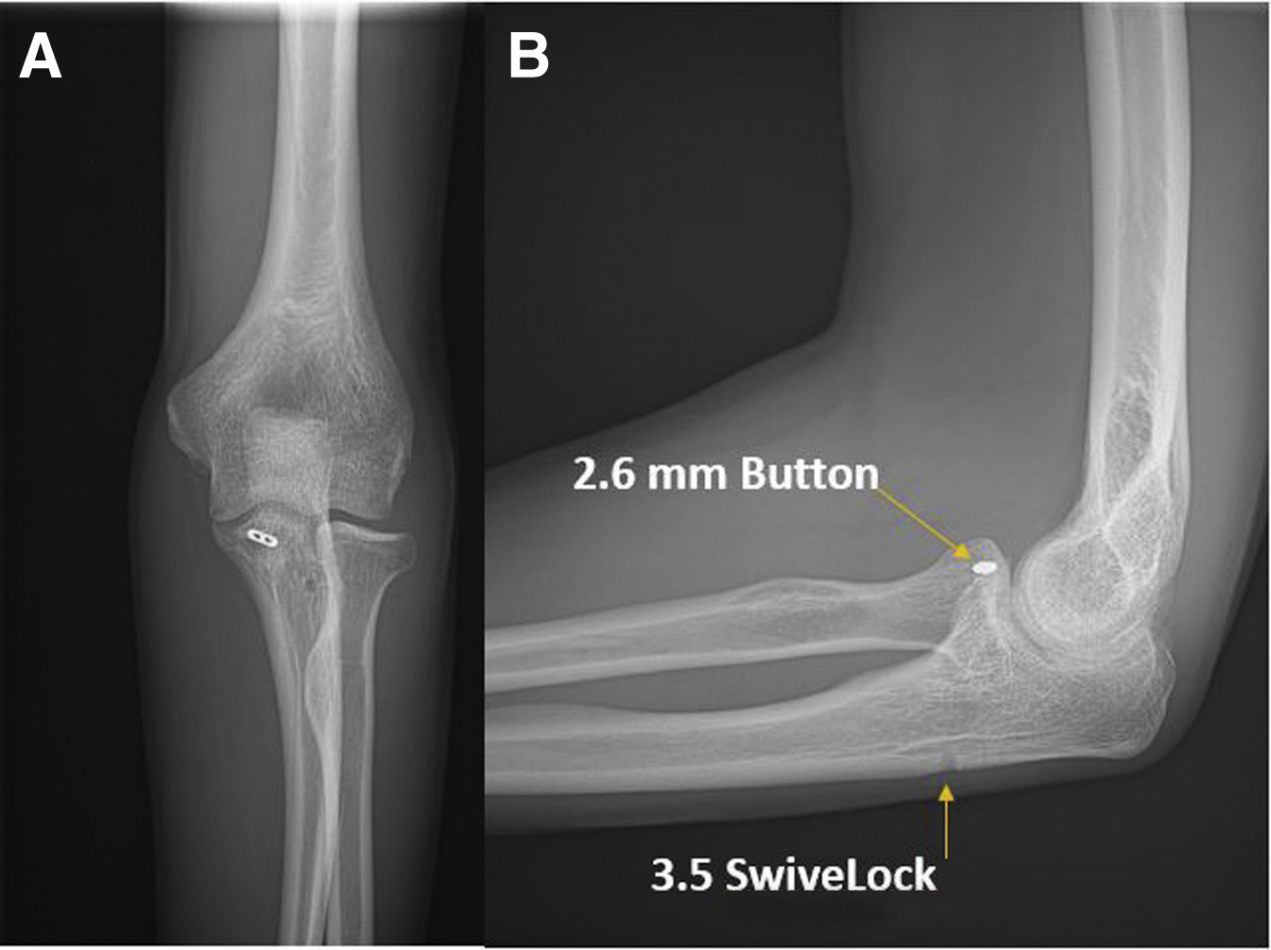
(A) Left elbow radiograph, anteroposterior view, demonstrates final fixation construct. (B) Left elbow radiograph, lateral view, demonstrating reduction of the coronoid fracture with suture button and knotless suture anchor and repair of the lateral collateral ligament complex. Note the posterior subluxation of the radial head has been corrected.

At this point, with the elbow in supination and the ligaments provisionally repaired, the radial head should point directly at the capitellum without any posterior subluxation. After imbrication of the ligament complex to the distal anchor, and reattachment of the extensor origins more proximally, the remaining muscular interval may be closed with interrupted absorbable braided sutures to ensure water-tight capsular closure. The elbow is taken through a complete range of motion and stability is confirmed in flexion, extension, pronation, and supination. The final construct may be confirmed fluoroscopically. Standard skin closure is then performed (Fig 5).

### Aftercare

Postoperatively, patients are placed in a posterior slab splint with the forearm in neutral to slight pronation. The splint is removed 10 to 14 days later at the first postoperative visit, and the patient is converted into a removable brace. In the absence of poor wound healing, progressive motion is then initiated under the guidance of an occupational therapist with a goal of full range of motion 8 to 10 weeks postoperatively. Strengthening is initiated at 12 weeks postoperatively, working from flexion and pronation (most stable) to supination and extension (least inherently stable). Full recovery is expected between 4.5 and 6 months from the time of surgery (Table 1).

**Table 1:** Pearls and Pitfalls

Pearls
• Use of a small-diameter shaver during fracture debridement/reduction will assist in joint expansion and allow the anterior capsule to float out of view.
• Use of an all-suture anchor device on the ulnar boarder can reduce symptomatic hardware.
• When working laterally, pronate the arm to move the PIN away from the operative field.
• Postoperative strengthening should start in a relative position of flexion/pronation, where the lateral complex is more stable.
Pitfalls
• Failure to identify the ulnar nerve and mark appropriate portals before arthroscopy can increase iatrogenic nerve injuries.
• Making your viewing portal in the standard PAMP position can result in difficult viewing, given the zero-degree scope.
• Overtensioning the lateral ligament complex can result in loss of range of motion/stiffness postoperatively.

PAMP, proximal anteromedial portal; PIN, posterior interosseous nerve.

## Discussion

Posterolateral rotatory instability has been described as a spectrum of injury resulting from failure of the soft-tissue and bony stabilizers of the elbow.^4^ These include the radial collateral ligament, the lateral ulnar collateral ligament, the annular ligament, the radial head, the coronoid process, as well as dynamic stabilizers such as the wrist flexors and extensors. Classically resulting from a fall on an outstretched hand, disruption of the lateral ligament complex, radial head, and/or coronoid process results from a valgus and rotatory load to the elbow. This spectrum of injury has been called the “terrible triad” due to difficulties with treatment and historically poor surgical outcomes. Nonoperative treatment is especially fraught with complications including pain, reduced function, and persistent instability.^5^ Surgical treatment has been shown to improve these outcomes and is recommended for many of these patients.

As arthroscopic technology has continued to improve, an increasing number of patients with posterolateral rotatory instability and terrible triad injuries are being treated, at least in part, using minimally invasive, arthroscopic techniques. A number of studies have shown comparable outcomes with arthroscopic techniques for ligament repair and/or reconstruction, as well as fixation of coronoid fractures, when compared with open techniques.^6^, ^7^, ^8^, ^9^ These techniques generally use standard elbow arthroscopic access to reduce and stabilize coronoid fractures or anterior capsular disruptions, resulting in long-term elbow stability with direct visualization of joint reduction and reduced soft-tissue dissection.

While traditional arthroscopy has been shown to be valuable in the treatment of elbow pathology, proximity of neurovascular structures, small intraarticular working space, and issues with fluid extravasation are problems that can make the procedure technically demanding. The role for small bore needle arthroscopy for both diagnostic and therapeutic applications is becoming clearer in the literature.^1^, ^2^, ^3^ NA has been used in the diagnosis of idiopathic knee pain in the clinic setting and as a useful tool for diagnostic shoulder arthroscopy. Its advantages in terms of fluid management also have been described, possibly resulting in reduced extravasation and shorter operative times. All of these advantages are attractive in the treatment of intra-articular elbow pathology. We suggest adjunctive use of the small-bore arthroscope is particularly attractive in the setting of terrible triad injuries and their variants.

## Conclusions

NA is a useful adjunct to arthroscopic examination, debridement, reduction, and fixation of coronoid tip fractures, particularly in the setting of a largely intact radial head or minimally displaced radial neck fracture.

## Supplementary Datas

ICMJE author disclosure forms

Video 1 Details the described technique in a stepwise approach to arthroscopically aided fixation of the coronoid process, followed by open reconstruction of the lateral collateral ligament complex (Lateral decubitus position with left elbow with anterior medial viewing portal).
